# A method for determining haploid and triploid genotypes and their association with vascular phenotypes in Williams syndrome and 7q11.23 duplication syndrome

**DOI:** 10.1186/s12881-018-0563-3

**Published:** 2018-04-04

**Authors:** Michael D. Gregory, Bhaskar Kolachana, Yin Yao, Tiffany Nash, Dwight Dickinson, Daniel P. Eisenberg, Carolyn B. Mervis, Karen F. Berman

**Affiliations:** 1 Section on Integrative Neuroimaging, Clinical and Translational Neuroscience Branch, National Institute of Mental Health, National Institutes of Health, 10 Center Drive 3C-216, Bethesda, MD 20892 USA; 2 Human Brain Collection Core, National Institute of Mental Health, National Institutes of Health, Bethesda, MD USA; 3 Statistical Genomics Core, National Institute of Mental Health, National Institutes of Health, Bethesda, MD USA; 4 Neurodevelopmental Sciences Laboratory, Department of Psychological & Brain Sciences, University of Louisville, Louisville, KY USA

**Keywords:** CNV, Haploid, Triploid, Williams syndrome, Dup7, SVAS, Elastin, PennCNV, Aortic dilation, Genotype-phenotype association

## Abstract

**Background:**

Williams syndrome ([WS], 7q11.23 hemideletion) and 7q11.23 duplication syndrome (Dup7) show contrasting syndromic symptoms. However, within each group there is considerable interindividual variability in the degree to which these phenotypes are expressed. Though software exists to identify areas of copy number variation (CNV) from commonly-available SNP-chip data, this software does not provide non-diploid genotypes in CNV regions. Here, we describe a method for identifying haploid and triploid genotypes in CNV regions, and then, as a proof-of-concept for applying this information to explain clinical variability, we test for genotype-phenotype associations.

**Methods:**

Blood samples for 25 individuals with WS and 13 individuals with Dup7 were genotyped with Illumina-HumanOmni5M SNP-chips. PennCNV and in-house code were used to make genotype calls for each SNP in the 7q11.23 locus. We tested for association between the presence of aortic arteriopathy and genotypes of the remaining (haploid in WS) or duplicated (triploid in Dup7) alleles.

**Results:**

Haploid calls in the 7q11.23 region were made for 99.0% of SNPs in the WS group, and triploid calls for 98.8% of SNPs in those with Dup7. The G allele of SNP rs2528795 in the *ELN* gene was associated with aortic stenosis in WS participants (*p* < 0.0049) while the A allele of the same SNP was associated with aortic dilation in Dup7.

**Conclusions:**

Commonly available SNP-chip information can be used to make haploid and triploid calls in individuals with CNVs and then to relate variability in specific genes to variability in syndromic phenotypes, as demonstrated here using aortic arteriopathy. This work sets the stage for similar genotype-phenotype analyses in CNVs where phenotypes may be more complex and/or where there is less information about genetic mechanisms.

**Electronic supplementary material:**

The online version of this article (10.1186/s12881-018-0563-3) contains supplementary material, which is available to authorized users.

## Background

Williams syndrome ([WS], MIM194050) and the reciprocal genetic disorder, 7q11.23 duplication ([Dup7], MIM609757), are caused by hemideletion or duplication, respectively, of approximately 1.6 megabases on chromosome 7q11.23 [1]. These disorders are associated with distinctive phenotypes, including contrasting neurobehavioral strengths and weaknesses. Individuals with WS, having one copy of some 26 affected 7q11.23 genes, are typically characterized by a hypersocialiality (social disinhibition with increased social drive), significant nonsocial anxiety, and a cognitive profile of impaired visuospatial construction abilities, and relatively preserved language skills [2]. Interestingly, individuals with Dup7, in whom the same set of genes are duplicated [3], show the opposite pattern: impaired social functioning with high social anxiety, preserved visuospatial abilities, and speech delay or disorde [1, 3]. Additionally, people with these 7q11.23 copy-number variations (CNVs) show contrasting cardiovascular abnormalities: Individuals with WS frequently have stenotic lesions, such as supravalvular aortic stenosis ([SVAS], MIM185500), which often come to clinical attention perinatally and may require surgical correction [1, 4]. In contrast, Dup7 is associated with dilation of the ascending aorta and aortic arch [5–7].

**Fig. 1:**
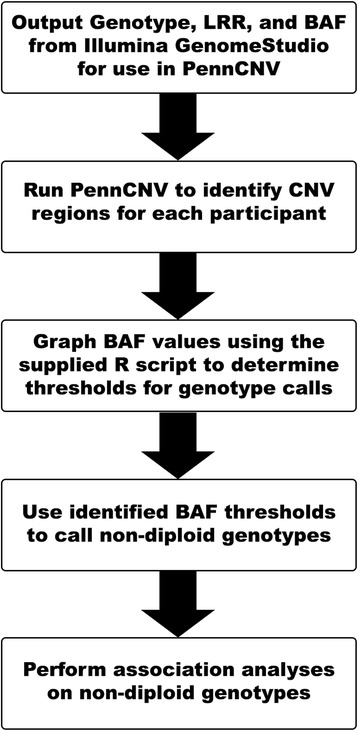
Flowsheet describing the pipeline to identify non-diploid genotypes and perform association analyses in CNV regions

The well-demarcated genetics and opposing phenotypes in these disorders offer opportunities to investigate gene-dosage effects, to understand how specific genetic mechanisms are translated into individual clinical presentations, and to test methods for determining genotypes in CNVs [8]. Though persons with WS and Dup7 generally exhibit the contrasting phenotypes described above, within each group there is considerable interindividual variability in the degree phenotypes are expressed [3, 9]. For instance, not all individuals with 7q11.23 CNVs manifest aortic disease. One explanation may be that sequence variation of the remaining (in WS) or duplicated (in Dup7) alleles within 7q11.23 causes variability in gene functioning, which may, in turn, impact symptom severity. Available software, such as PennCNV, has been used to identify CNVs using commonly-acquired SNP-chip data. However, using this same chip data to also determine the underlying non-diploid genotypes of CNV regions has not been done. Here, we describe a method for using these commonly-acquired SNP-chip data to identify the remaining or duplicated alleles in CNV regions, and apply it to participants with known CNVs of the 7q11.23 WS locus (Fig. 1).

Further, as a proof-of-concept, we tested for associations of data obtained in this manner with the penetrance of aortic pathology in WS and Dup7. We chose arteriopathy as a phenotype-of-interest because there is substantial a priori evidence implicating a particular gene in the 7q11.23 WS locus, namely *elastin* (*ELN*); hemideletions, translocations, gross deletions, and point mutations of *ELN* alone, can cause SVAS in an autosomal dominant fashion in individuals who do not have WS [4, 10]. We first conducted a region-wide association study in WS, expecting *ELN* sequence variation to be associated with SVAS penetrance. As a further test, we carried forward identified SVAS-associated SNPs for combined-group (WS and Dup7) analysis, hypothesizing that the SVAS-associated risk alleles would show opposing (i.e. protective) effects for aortic dilation in Dup7.

## Methods

### Participants

Twenty-five children known to have classic WS deletions (mean age = 10.5 ± 4.4, 17 girls) and 13 children with Dup7 (mean age = 12.4 ± 3.1, six girls) participated in a larger investigation of brain and behavior associated with 7q11.23 CNVs at the National Institutes of Health (NIH) Clinical Center. Parents provided written informed consent and children provided assent, as approved by the NIH Combined Neurosciences IRB. Participants underwent comprehensive physical examination and detailed medical chart review by a licensed physician.

### Genetic analyses: Determining regions of copy number variation (CNV)

**Fig. 2:**
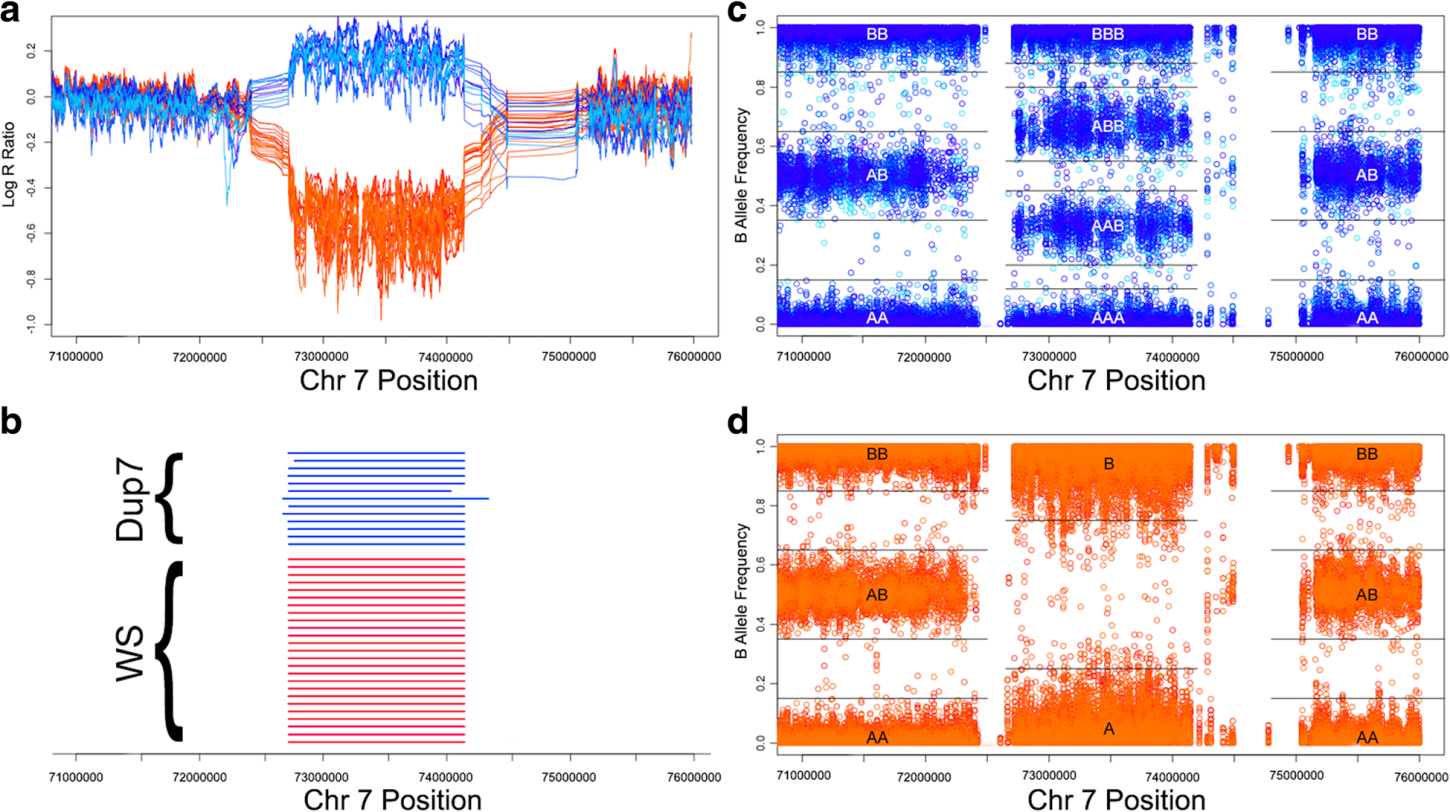
Method used to make haploid and triploid calls in the 7q11.23 critical region. Panel (**a**) shows the probe intensity values (Log R Ratio; LRR) across the 7q11.23 locus for all participants. Note the increase in LRR in participants with known duplications (blues) and the decrease in LRR in participants with known deletions (oranges). Panel (**b**) shows the chromosomal locations of called duplications (blues) and deletions (oranges) in this region for all participants. Panels (**c** and **d**) show the B allele frequency for each 7q11.23 SNP for all participants with duplications (Panel (**c**), blue colors) or deletions (Panel (**d**), orange colors); black lines represent thresholds used to make genotype calls, and overlying letters represent genotypes. Varying shades of blue and orange represent individual participants

Participants provided blood samples which were genotyped using Illumina HumanOmni5-4v1.1 SNP-chips. Probe intensity values (Log R ratios [LRR]) and B-allele frequency (BAF) values were extracted for each SNP using Illumina’s GenomeStudio version 2.0. PennCNV version 1.03 [11] was used to delineate areas of CNV for each individual, while controlling for the GC content of the genetic region [12]. Only CNVs that had at least 10 consecutive SNPs and were at least 10 kB in length were carried forward in the analysis. Additionally, regions identified as CNVs within 1 kb of each other were merged (Fig. 2).

### Genetic analyses: Determining non-diploid genotypes for each SNP

Further analysis of CNVs was restricted to the 7q11.23 WS region. Using R scripts developed in-house (available as an Additional file 1), we sought to identify haploid (for participants with WS) and triploid (for participants with Dup7) genotypes for each 7q11.23 SNP. BAF plots for all SNPs in the 7q11.23 locus were visually-examined to determine fixed thresholds for each genotype. For our sample, the thresholds used for hemideletions were A = 0–0.25 and B = 0.75–1. For Dup7, the thresholds were AAA = 0–0.12, AAB = 0.2–0.45, ABB = 0.55–0.8, BBB = 0.88–1. These thresholds were then applied to determine the underlying haploid or triploid genotypes: A or B genotypes for each SNP in individuals with hemideletions; or AAA, AAB, ABB or BBB for each SNP in individuals with duplications (Fig. 2c and d).

### Genotype-phenotype association analyses

After determining CNV genotypes for each SNP, we tested our methods by searching the 7q11.23 WS region for associations of these SNPs with SVAS severity in our WS sample. SVAS severity was determined via a detailed chart review of available medical records by a physician. Persons with WS who required surgery to correct SVAS were categorized as having severe SVAS (8/25 WS patients), and those who did not have surgery were categorized as having mild or absent (17/25 WS patients), providing a categorical phenotype for association analyses. Chi-squared tests of the association between the degree of SVAS with every SNP in 7q11.23 genes were performed using R (code provided as an Additional file 1). SNP-level statistics were Bonferroni-corrected for multiple comparisons based on the effective number of LD-independent SNPs), as determined by GEC software version 0.2 [13]: within the *ELN* gene given the substantial a priori evidence implicating this gene in SVAS pathology (5.35 LD-independent SNPs, p_uncorrected_ < 0.0094 = p_Bonferroni_ < 0.05) and within the 7q11.23 WS locus for SNPs in other genes (112 LD-independent SNPs, p_uncorrected_ < 4.46 × 10^− 4^ = p_Bonferroni_ < 0.05. Significant results in our haploid WS group were then further tested in our smaller Dup7 sample.

For individuals with Dup7, the presence or absence of aortic dilation was similarly determined by medical chart review (4/13 Dup7 patients with aortic dilation). For SNPs found to be significantly related to SVAS in persons with WS, we used logistic regression to predict aortic arteriopathy based on the interaction between diagnosis (WS or Dup7) and SNP genotype. Because the phenotype in Dup7 is opposite to that in WS (dilation vs. stenosis), we expected the risk alleles at identified SNPs to be opposite in the two CNV groups.

## Results

CNVs were identified by PennCNV in the 7q11.23 locus for all individuals (Fig. 2). Start and stop locations for deletions in this locus were nearly identical across people with WS, consistent with prior literature showing stereotyped deletions in nearly 95% of people with WS [14]. Duplications in this locus were identified for all individuals with known Dup7, although variability in the endpoints was slightly greater than in WS.

**Fig. 3:**
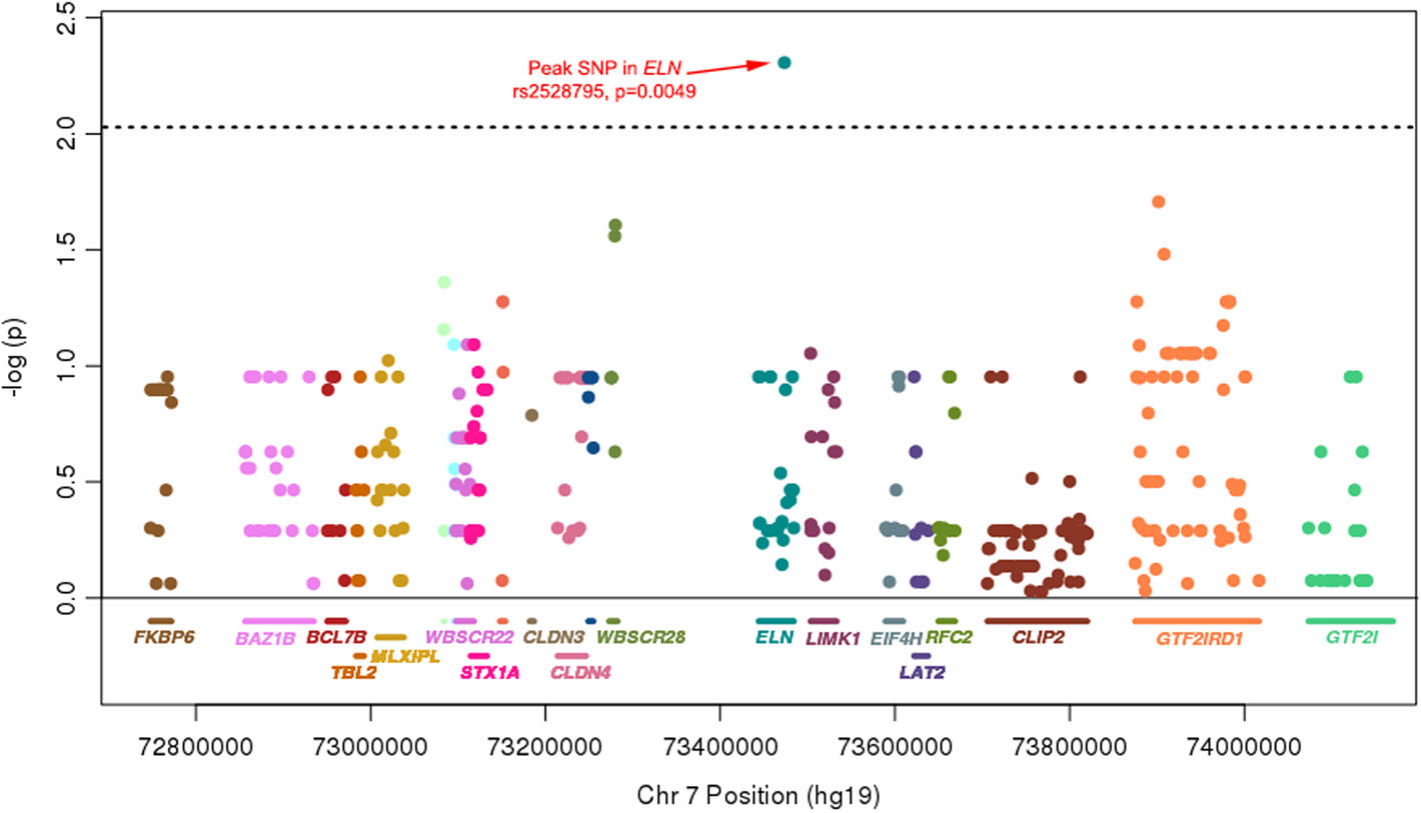
SNP associations with severity of cardiovascular symptoms in the 7q11.23 region. A Manhattan plot of SNP associations with the severity of supravalvular aortic stenosis in participants with WS, across the WS critical region. SNPs are colored by their respective genes, which are shown on the X-axis. Note that as expected, the peak SNP (rs2528795) lies in the *ELN* gene. Dashed line indicates significance level, correcting for the number of SNPs within the a priori defined *ELN* gene

Using BAF thresholds (Fig. 2), haploid calls were made for 99.0% of SNPs in participants with WS (38,105/38500) and triploid calls in 98.8% of SNPs in participants with Dup7 (19,782/20020 SNPs). First, in participants with WS, we found that within remaining, haploid alleles, the peak association with severity of SVAS was located in a SNP in the *ELN* gene (rs2528795, p_uncorrected_ = 0.0049, p_Bonferroni_ = 0.026, Fig. 3), consistent with a priori evidence and predictions. No SNPs in other genes showed significant association after correcting for multiple comparisons across the 7q11.23 WS locus.

**Fig. 4:**
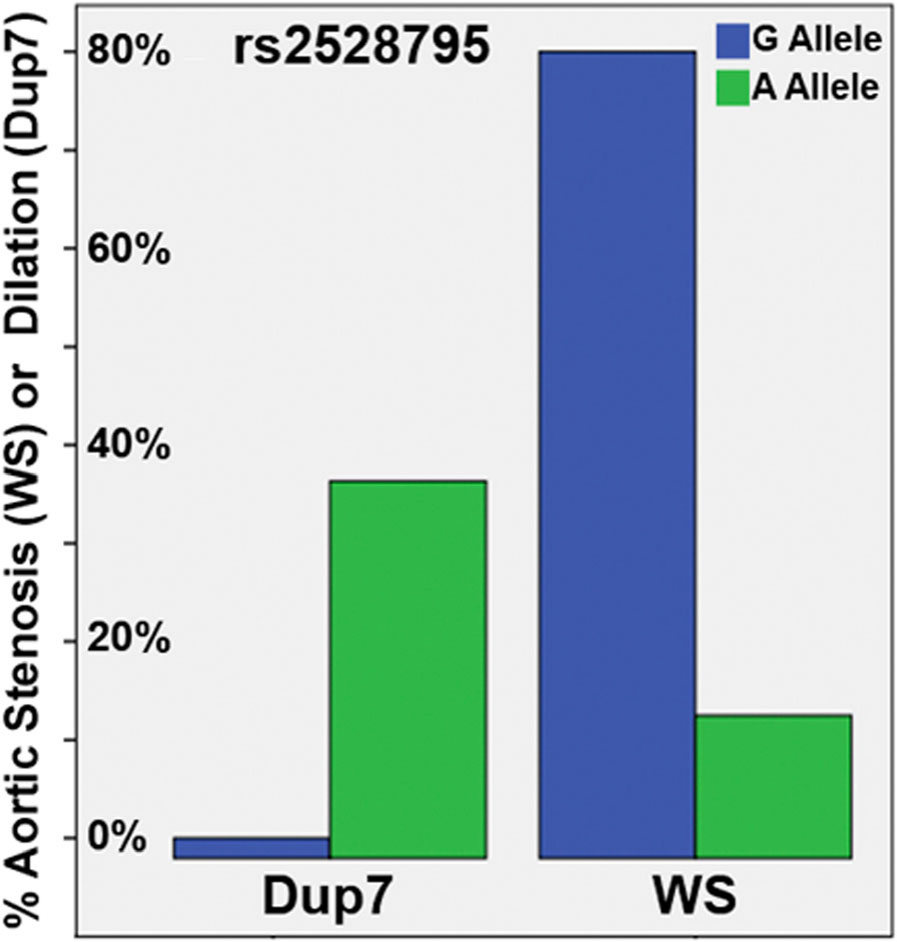
Associations of aortic pathology with rs2528795 genotype in 7q11.23 WS region deletions and duplications. Figure shows the percentage of severe SVAS (in participants with WS, left) and aortic dilation (in participants with Dup7, right) by rs2528795 genotype. Note that the G allele, which is the risk allele for stenosis in WS, is protective for aortic dilation in Dup7 (the opposite phenotype); the interaction of diagnosis and genotype predicted participants’ cardiovascular status, with over one-third of the variance explained by the model (Nagelkerke’s R^2^ = 0.351, *p* < 0.021)

Next, testing for effects of *ELN* rs2528795 SNP in both WS and Dup7, we found the interaction of diagnosis and rs2528795 genotype significantly predicted participants’ cardiovascular status, explaining over one-third of the variance in arteriopathy (Nagelkerke’s R^2^ = 0.351, *p* < 0.021). For individuals with WS, 80% of those with the *ELN* rs2528795 G allele had severe SVAS, whereas 84% of those with the A allele did not. In contrast, for individuals with Dup7, 40% of those with at least 2 copies of the A allele had aortic dilation, while no participants (0%) with at least two copies of the G allele had aortic dilation (Fig. 4). In other words, in the context of WS, the rs2528795 G allele (or a genetic signal in linkage with it) increases the severity of SVAS, whereas in Dup7 the same allele is protective against aortic dilation.

## Discussion

Here, we describe a pipeline for using LRR and BAF values from commonly available, genome-wide SNP-chip data, to determine the underlying genotype of haploid or triploid alleles in CNV regions. In patient populations with syndromic CNVs, such as WS and Dup7, this method can help to uncover relationships between individual genes and variation in expression of associated phenotypes, as shown here for aortic arteriopathy.

In making the CNV calls, we found that initiation and stop sites of hemideletions were nearly identical for all participants with WS, in line with prior literature showing that, due to sequence homology flanking the WS critical region, 95% of people with WS have stereotyped hemideletions [14]. In Dup7, though the start/stop sites of the duplications were similar, there was nominally more variability across individuals than was seen in WS. It is possible that this observation does not reflect true copy number variation, but, instead, is related to the methods employed by PennCNV. As seen in Fig. 2, the magnitude of the increase in LRR of duplicated regions (blue lines) is less than the magnitude of the decrease in hemideleted regions (orange lines), consistent with the fact that the exponential of LRR increases linearly with copy number [11]. Thus, it is possible that called duplications may be more susceptible to small errors than deletions. However, it is also possible that more variability exists in the start/end points of 7q11.23 duplications, perhaps due to greater chromosomal instability during replication when an extra copy is introduced. Future work using sequencing data may be valuable in further examining this possibility.

In our samples, the call rate for non-diploid genotypes in the 7q11.23 WS locus was 99%, which is similar to those reported for diploid calls in other regions (97.9%–99.9%) using Illumina BeadArray chips [15]. There are multiple potential sources for non-called or miscalled SNPs using SNP-chips. As described by Pompanon et al., these may include DNA sample quality, interactions between DNA molecules, biochemical causes, or human error [16]. Despite these potential errors, the genotyping done here is of similar quality to that routinely performed in diploid regions.

Our findings regarding *ELN* support the use of the pipeline developed here, using commonly-available SNP data to test for genotype-phenotype associations within 7q11.23 and other CNVs. We found that in the context of Dup7, the G allele of rs2528795 in *ELN* is protective against aortic dilation, but in the context of WS, the same allele increases risk of aortic stenosis. The inverse directionality of risk alleles in the WS and Dup7 groups, along with the consistency of these findings with our hypotheses and prior evidence that *ELN* is implicated in cardiovascular abnormalities, are a positive initial test of this method. While it is known that mutations of *ELN* can cause non-syndromic SVAS in an autosomal dominant fashion [4, 10], the interindividual variation in the expressivity and penetrance of SVAS in WS has not been fully explained. Our findings add to previously published exon sequence data that describe relationships between *ELN* variation and cardiovascular phenotypes in WS [15]. Our methodology, relying on the application of easily obtained chip-based SNP information, may make similar investigations easier to perform.

Further, though it has been hypothesized that involvement of *ELN* underlies aortic dilation found in some individuals with Dup7 [6, 17], there is little established evidence of this association. While our sample size was too small to identify a significant effect when examining rs2528795 in Dup7 alone, we did find a significant interaction of genotype-by-diagnosis on aortic status (dilation vs. stenosis) when considering both patient groups together, suggesting that *ELN* sequence variation is indeed related to dilation in Dup7. These findings are supported by considerable a priori evidence: the elastin protein is a biopolymer and a critical component of the extracellular matrix, constituting nearly 30% of the aorta [18]. It is formed by crosslinking precursor tropoelastin molecules, the gene product of *ELN* [19], and the concentration of elastin is increased in aortic dilations [20]. While interactions of *ELN* variation with genetic variation in other 7q11.23 genes, and throughout the genome, undoubtedly impact expression of arteriopathy, our results support the use of the method developed here to uncover genotype-phenotype links in individuals with CNVs.

As considerable variability exists in the expression of phenotypes caused by 7q11.23 CNVs (as well as other CNVs), one potential explanation, which is tested here, is that variability is due to sequence variation within the affected genomic region. However, there may be other causes for this phenotypic variability. For example, genetic variation outside of the CNV regions of interest, including other SNPs or CNVs, may also impact these phenotypes. Additionally, environmental factors may also play important roles. Though there is significant a priori evidence implicating *ELN* with aortic pathology, the SNP identified here, rs2528795, has not been previously linked to SVAS or aortic dilation. However, one prior study found weak associations between this SNP and autism [21], though *ELN* has minimal expression in the human brain [22]. Similarly, a related phenotype, aortic root diameter, has not been previously associated with the variation at the 7q11.23 locus in prior studies [23, 24], despite the known pathology found in individuals with these CNVs.

## Conclusions

In summary, we present a method to make genotype calls in individuals with syndromic CNVs. Additionally, using the well-established genotype-phenotype link between *ELN* and aortic arteriopathy, we show that variability in remaining or duplicated alleles in the 7q11.23 CNV regions, as identified in this manner, can be associated with the severity of phenotype expression. This work provides support for applying this approach to uncover novel genetic associations with phenotypes where the clinical presentation is more complex, such as cognitive and brain-based features, and/or where there is less information about causative genes.

## Additional file

Additional file 1: R script used for analyses. This file contains R code used to identify CNV genotypes and to perform association analyses with haploid genotypes. (R 4 kb)
